# True leukonychia as the presenting sign of early thromboangiitis obliterans

**DOI:** 10.1016/j.jdcr.2024.01.001

**Published:** 2024-01-13

**Authors:** Mohammed S. Hamid, Jane Harrell, Julie E. Mervak

**Affiliations:** a University of Michigan Medical School, Ann Arbor, Michigan; b Department of Dermatology, University of Michigan, Ann Arbor, Michigan

**Keywords:** Buerger disease, leukonychia, nail, onychodystrophy, smoking, thromboangiitis obliterans, true leukonychia, vascular insufficiency, white nails

## Introduction

Leukonychia, white discoloration of nails, has a broad range of etiologies with the diagnosis and management based on monodactylous vs polydactylous presentation and clinical determination of true vs apparent leukonychia.^1^ True leukonychia represents an abnormality in the matrix and nail plate with no fading of the white color with pressure. This occurs due to abnormal distal matrix keratinization leading to persistent parakeratosis and keratohyalin granules in the intermediate and ventral nail plates.^1^ In contrast, apparent leukonychia represents a nail bed abnormality and fades with pressure due to reduction in nail bed edema and improved visualization of nail bed vasculature. Pseudoleukonychia, which does not fade with pressure, describes white color on the superficial nail plate, most often secondary to damage from nail cosmetics or superficial white onychomycosis.^1^ Leukonychia is further categorized as punctate, transverse, longitudinal, or partial/total. Punctate or transverse true leukonychia is frequently due to trauma, but other etiologies should be considered.^1^ Partial/total true leukonychia involving all digits can be seen in selenium deficiency, hydroxyurea exposure, or rare genodermatoses.^1^ Isolation of partial/total true leukonychia to a few digits should prompt evaluation for neurovascular disorders, such as reflex sympathetic dystrophy or peripheral neuropathy.^1^ In this report, we describe a case of true leukonychia isolated to the right second and third fingernails as the presenting sign of early thromboangiitis obliterans (TAO).

## Case report

**Fig 1:**
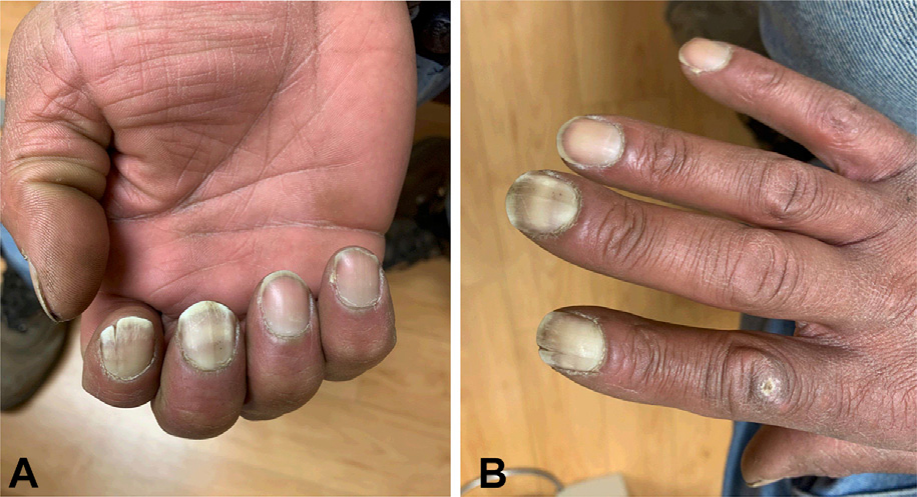
**A** and **B**, True leukonychia of the right second and third fingernails with mild edema at the proximal nailfold and distal fingertip. Distal longitudinal split of the right second fingernail. Subtle gray-brown longitudinal melanonychia of the right third fingernail most consistent with melanocyte activation in patient of skin of color.

A 43-year-old man with left-sided cerebral palsy, resulting in weakness of his left upper extremity since birth, was referred to dermatology for evaluation of a 5-year history of onychodystrophy of the right second and third fingernails. Prior to referral, he was treated empirically for onychomycosis with 3 months of terbinafine without resolution. He denied positional pain, muscle weakness, or color change consistent with Raynaud phenomenon. He noted progressive cold sensitivity and numbness with cold exposure of the affected fingers. He was a current smoker with a 9 pack-year history. He was not taking any medications. Physical examination revealed true leukonychia on the right second and third fingernails with signs of early ventral pterygium, distal nail plate split of the second fingernail, light brown longitudinal melanonychia of the third fingernail consistent with melanocyte activation, and subtle edema of the proximal nailfolds and distal fingertips (Figs 1, *A* and *B*; and 2). Radial and ulnar pulses were palpable. Phalen maneuver, to assess for carpal tunnel syndrome (CTS), was negative. Nail plate clipping from the right second fingernail showed ventral surface parakeratosis consistent with true leukonychia and negative Grocott methenamine silver stain for hyphae, ruling out onychomycosis. Given concerns for TAO, he was referred to cardiology. On examination, Allen test was positive, indicative of vascular occlusion distal to the wrist. An upper extremity arterial doppler ultrasound, computed tomography angiography with upper extremity runoff, and wrist-brachial indices showed no evidence of large vessel arterial disease. Abnormalities in the small digital arteries could not be excluded and angiography was discussed; however deferred because of high clinical suspicion for TAO and unlikely event that additional invasive testing would change management. Smoking cession was reviewed and strongly encouraged.

**Fig 2:**
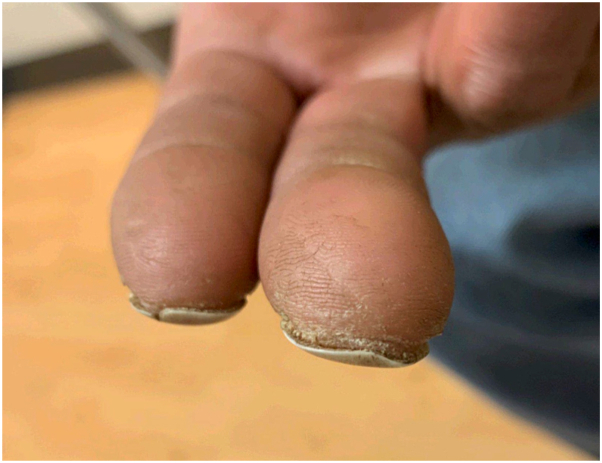
Signs of early ventral pterygium of right second and third fingernails suggestive of vascular insufficiency.

## Discussion

TAO, also known as Buerger disease, is a nonatherosclerotic inflammatory disorder that affects the small and medium-sized arteries of the distal extremities initially presenting as episodic pain and coldness of the digits secondary to digital ischemia.^2^ This can progress to claudication, ulceration, and need for amputation.^2^ Although the pathogenesis is not fully understood, TAO is most often seen in men aged <50 years and is strongly associated with the use of tobacco products.^3^ A normal wrist-brachial index does not rule out TAO given the distal vascular involvement. Positive Allen test in a young smoker is concerning for TAO.^4^

Although a patient with TAO may present to dermatology at later stage with digit ulceration and necrosis, an astute assessment of the nails can lead to early diagnosis. Early digital ischemia presents with slowed nail growth resulting in nail plate thickness and roughness with transverse or longitudinal ridging, splinter hemorrhages, and onychogryphosis.^5^ At later stages, nail changes include Beau’s lines, onychomadesis, or permanent dystrophy, including potential permanent nail loss.^5^ A recent report described 2 cases of TAO nail disease: one with chronic paronychia of the second and third fingernails with proximal leukonychia and distal hemorrhagic lesion of the third fingernail; the other with onycholysis, nail bed erosion, and chronic paronychia of the second and third fingernails.^5^ Interestingly, as seen in our case, these patients had involvement of fingers used to hold a cigarette when smoking. It is postulated that vascular insufficiency leads to nail matrix damage and subsequent keratinization abnormalities presenting as leukonychia.^5^

True leukonychia isolated to a few digits should prompt evaluation for neurovascular insufficiency, including reflex sympathetic dystrophy and CTS.^1^^,^^6^ CTS can present with second and third fingernail changes, including very rarely leukonychia.^6^ Although CTS was considered in our patient, smoking history, lack of positional pain, lack of paresthesia at night, negative Phalen maneuver, and cardiology examination findings pointed away from this diagnosis. Isolated true leukonychia in a smoker should prompt discussion about importance of smoking cessation and risks of progression and even potential need for amputation should smoking continue. Although further treatment options are limited given small caliber of vessels involved, evaluation by cardiology or vascular surgery could be considered.

## Conflicts of interest

None disclosed.
